# Modulation of Macrophage Responses by CMX, a Fusion Protein Composed of Ag85c, MPT51, and HspX from *Mycobacterium tuberculosis*

**DOI:** 10.3389/fmicb.2017.00623

**Published:** 2017-04-12

**Authors:** Adeliane C. da Costa, Danilo P. de Resende, Bruno de P. O. Santos, Karina F. Zoccal, Lúcia H. Faccioli, André Kipnis, Ana P. Junqueira-Kipnis

**Affiliations:** ^1^Laboratory of Immunopathology of Infectious Disease, Department of Microbiology, Immunology, Parasitology and Pathology, Tropical Institute of Pathology and Public Health, Federal University of GoiásGoiânia, Brazil; ^2^Laboratory of Inflammation and Immunology of Parasitoses, Department of Clinical, Toxicological and Bromatological Analyses, School of Pharmaceutical Sciences of Ribeirão Preto, University of São PauloSão Paulo, Brazil; ^3^Laboratory of Molecular Bacteriology, Department of Microbiology, Immunology, Parasitology and Pathology, Tropical Institute of Pathology and Public Health, Federal University of GoiásGoiânia, Brazil

**Keywords:** recombinant vaccine, tuberculosis, BCG, cytokines, TLR-2, TLR-4

## Abstract

*Mycobacterium bovis* Bacillus Calmette–Guérin (BCG) is a vaccine used to prevent tuberculosis (TB). Due to the poor protection conferred by BCG in adults, new, more effective formulations have been developed. A recombinant BCG vaccine expressing the CMX fusion protein Ag85c_MPT51_HspX (rBCG-CMX) induced Th1 and Th17 responses and provided better protection than BCG. It has been shown that *Mycobacterium smegmatis* expressing CMX also induces better protection than BCG and is a strong macrophage activator. The aim of the present study was to evaluate macrophage activation by the recombinant CMX fusion protein and by rBCG-CMX and to evaluate their ability to generate vaccine-specific immune responses. The results demonstrate that rCMX protein expressed by BCG (rBCG-CMX) activates pulmonary macrophages; increases the expression of activation molecules, cytokines, and MHC-II. The interaction with rCMX activates the transcription factor NF-κB and induces the production of the cytokines TGF-β, TNF-α, and IL-6. The *in vitro* stimulation of bone marrow-derived macrophages (BMMs) from TLR-4 or TLR-2 KO mice showed that in the absence of TLR-4, IL-6 was not produced. rBCG-CMX was unable to induce CMX-specific Th1 and Th17 cells in TLR-4 and TLR-2 KO mice, suggesting that these receptors participate in their induction. We concluded that both the rBCG-CMX vaccine and the rCMX protein can activate macrophages and favor the specific immune response necessary for this vaccine.

## Introduction

Macrophages are important components of the innate immune response to tuberculosis (TB) because they directly participate in the response to *Mycobacterium tuberculosis* (Mtb) (Lang, 2013). Mtb is an intracellular pathogen that interacts with macrophages and dendritic cells via receptors such as toll-like receptors (TLRs) (Rivera-Marrero et al., 2002; Bafica et al., 2005). TLRs are membrane receptors that interact with conserved molecules from pathogens to promote signal transduction that generates pro-inflammatory cytokines. When recognized by phagocytes, Mtb induces the production of cytokines, including TNF-α, IL-6, IL-12, IL-1α, and TGF-β (Bafica et al., 2005; Chatterjee et al., 2011; Huaux et al., 2015). Some Mtb proteins, such as ESAT-6, PPE57, Ag85c, and Rv0652, are recognized by TLR-2, CR3, and TLR-4 and modulate macrophage responses (Hetland and Wiker, 1994; Kim et al., 2012; Wang et al., 2012; Xu et al., 2015). ESAT-6, for example, has been shown to modulate macrophages and dendritic cells *in vitro*, producing cytokines that inhibit Th1 responses and that facilitate Th17 responses (Wang et al., 2012).

The BCG (Bacillus Calmette-Guérin) vaccine, which is the current vaccine used to control TB, is believed to induce a strong Th1 response but a much weaker Th17 response (Garcia-Pelayo et al., 2015). However, it has been shown that earlier Th17 responses to BCG are crucial to generate Th1 responses to this vaccine (Gopal et al., 2012). BCG is an attenuated strain of *Mycobacterium bovis*, which, during the attenuation process, lost important virulence regions (Cole et al., 1998). Despite being the only vaccine approved for human use and providing protection against TB meningitis and miliary TB in children, the protective effect of BCG is limited because it does not protect adults against pulmonary TB (Abubakar et al., 2013). This lack of protection in the adult phase may be due to its poor ability to induce a good vaccine response with a balance between Th1 and Th17 responses.

The induction of vaccine responses in mucosa may promote the induction of Th17 because mucosal macrophages have an anti-inflammatory profile, and they have the ability to produce both TGF-β and IL-6 (Linehan et al., 2015). The induction of macrophage apoptotic cell death can promote the release of apoptotic bodies that could induce the cross-presentation of vaccine peptides by major histocompatibility complex class I (MHC-I) and class II (MHC-II) molecules on dendritic cells, promoting the induction of mixed CD4^++^ T and CD8^+^ T responses (Farinacci et al., 2012).

The BCG vaccine expressing the CMX fusion protein (rBCG-CMX) and the mc^2^-CMX vaccine have been able to induce Th1 responses and potentiate the induction of Th17, thereby promoting an equilibrium in the induction of these cellular responses. Regardless of the vehicle used, BCG or *Mycobacterium smegmatis*, vaccines expressing the recombinant CMX protein (rCMX) have contributed to protection against Mtb. Moreover, when used in another vector, IKE-CMX, rCMX has been able to activate more macrophages than the vector alone (Junqueira-Kipnis et al., 2013; da Costa et al., 2014). Thus, macrophages appear to be involved in the protection conferred by the vaccine, indicating that rCMX may be modulating the innate immune response and promoting the vaccine immune response. The rCMX fusion protein is composed of immunodominant epitopes of the antigens rAg85c, rMPT51, and full-length rHspX (de Sousa et al., 2012). Ag85c (Rv0129c) and MPT51 (Rv3803c) are part of the same complex and are important virulence factors (Ohara et al., 1997). Ag85c, for example, participates in the synthesis of mycolic acid, a component of the Mtb cell wall, which represents more than 40% of the dry weight of Mtb (Harth et al., 1997; Kitaura et al., 2000; Sanki et al., 2009). The HspX antigen (Rv2031c) is an important protein for Mtb growth within macrophages (Yuan et al., 1998).

Based on this evidence, the aim of the present study is to evaluate whether the rCMX protein or BCG expressing CMX acts on macrophages to promote the vaccine immune response.

## Materials and Methods

### Animals

Specific pathogen-free 4–8-week-old BALB/c, C57BL/6, TLR-4^-/-^, and TLR-2^-/-^ mice were obtained from the School of Pharmacy, University of São Paulo (Universidade de São Paulo – USP). They were originally donated by S. Akira (Osaka University, Osaka, Japan). Animal housing for all experimental procedures consisted of a constant temperature (24 ± 1°C) and humidity (50 ± 5%) environment in HEPA-filtered isolators. The animals were fed a sterile diet specific to mice and were provided water *ad libitum* under controlled light conditions (12-h light and 12-h dark period). Paper nesting was provided weekly to enrich the animal environment. Animals were monitored daily for any symptoms of clinical disease or change in behavior by an attending veterinarian. Euthanasia was performed by cervical dislocation by a trained researcher. The animals were handled according to the guidelines of the Conselho Nacional de Controle e Experimentação Animal (CONCEA). The study was approved by the Ethics Committee for Animal Use (Comitê de Ética no Uso de Animais - CEUA; # 229/11) of the UFG.

### Antigens and Vaccines

The recombinant antigens of *Mycobacterium tuberculosis*, rAg85c and rCMX, were produced in *Escherichia coli* in the Immunopathology and Infectious Diseases Laboratory at the Institute of Tropical Pathology and Public Health (Instituto de Patologia Tropical e Saúde Pública - IPTSP), UFG. All antigens were prepared as previously described by de Sousa et al. (2012). After purification of the rAg85c and rCMX proteins, they were subjected to an LPS-removal process using a ToxinEraser^TM^ Endotoxin Removal Kit. The endotoxin levels were evaluated using the E-Toxate kit (Sigma) and did not exceed 0.03 endotoxin units. The procedure was performed according to the manufacturer’s instructions (GenScript – 860 Centennial Ave., Piscataway, NJ 08854, USA).

The strain of *M. bovis* BCG-Moreau, kindly donated by the Butantan Institute, was grown in 7H9 liquid culture medium supplemented with 10% oleic acid, dextrose, and catalase (OADC), 0.5% glycerol, and 0.05% Tween 80 and incubated at 37°C in a humidified atmosphere with 5% CO_2_ for approximately 21 days. The rBCG strains were obtained via the electroporation of the BCG-Moreau strain with the pLA71/CMX expression plasmid as described previously (da Costa et al., 2014).

### Murine Intranasal Infection with Empty BCG-pLA71 or rBCG-CMX

The BALB/c and C57BL/6 mice were divided into three groups: control (*n* = 5), empty BCG-pLA71 (*n* = 5), and rBCG-CMX (*n* = 5). Aliquots of the rBCG-CMX and empty BCG-pLA71 vaccines were removed from the -80°C freezer and diluted in 0.05% PBS Tween 80 at a concentration of 1x10^8^ CFU/mL. A total volume of 100 μL of the vaccine was intranasally administered in 20-μL doses, allowing the animal to breathe between doses. The saline group received 100 μL of PBS/0.05% Tween-80. The immunizations were performed in a single dose. After preparation of the vaccines, a sample was plated to confirm the concentration. After immunization, the animals were observed for 3 h to check for signs of apathy, wheezing, or any change in behavior indicating extreme discomfort. If an animal produced signs and symptoms that were incompatible with animal welfare, a trained veterinarian would have humanely euthanized the animal. No animals presented these symptoms during the experiment.

### Macrophages

Peritoneal and alveolar macrophages were obtained by peritoneal and bronchial alveolar lavage (Correa et al., 2014; da Costa et al., 2014), respectively. The alveolar lavages were centrifuged at 1,000 × *g* and 4°C for 10 min. The supernatant was discarded, and the cells were resuspended in 1 mL of complete RPMI medium (cRPMI – HIMEDIA, Mumbai, India) containing 2 mM-glutamine, 100 U/mL penicillin, 1000 U/mL streptomycin (GIBCO), 10 nM pyruvate, and 10% FBS. The cells were counted using Trypan blue (Code 1263C061, Amresco, Solon, OH, USA 44139-4300) in a hemacytometer. The peritoneal macrophage cultures were adjusted to a concentration of 10^6^ cells per well (24-well plates), whereas the alveolar macrophages were used at a concentration of 2 × 10^5^ cells per well (96-well plates). The plates were cultured for 24 h before stimulation with antigens. The recombinant antigens rAg85c and rCMX were used at a concentration of 20 μg/mL and were added to the macrophage cultures after standardization. After 24 h of stimulation, the culture supernatants were collected for cytokine analyses. The experiments were repeated three times in quadruplicate.

### Lung Homogenates

Four days after intranasal infection, the mice were euthanized by cervical dislocation. The left lung lobes were collected and prepared as described by da Costa et al. (2014). Once obtained, the lung was treated with a solution of DNAse IV (30 μg/mL; Sigma–Aldrich) and collagenase III (0.7 mg/ml; Sigma–Aldrich) for 1 h at 37°C. To obtain a cell suspension, the tissue was passed through a 70-μm cell filter (BD BioSciences, Lincoln Park, NJ, USA). The erythrocytes were lysed with lysis solution (0.15 M NH_4_Cl, 10 mM KHCO_3_), and the cells were then washed and resuspended in cRPMI medium and adjusted to 1 × 10^6^ cells/mL. The cell suspensions were divided for culture or flow cytometry. For culture, the cells were maintained for 24 h without stimulus and incubated at 37°C in a humidified 5% CO_2_ atmosphere. After this period, the culture supernatants were collected and stored at -20°C until the time of cytokine measurement. For flow cytometry, the cell suspensions were labeled immediately after isolation.

### Cytokine Analyses

The supernatants of the cell cultures stimulated with the rAg85c or rCMX proteins and supernatants of the cells infected with BCG, rBCG-CMX, or empty rBCG-pLA71 vaccines were used for IL-6 (limit of detection: 4 pg/mL), IL-1α (limit of detection: 6 pg/mL), and TGF-β (limit of detection: 8 pg/mL) analyses using the mouse IL-6, IL-1α, and TGF-β ELISA Ready-SET-Go (eBioscience, Inc.) kits according to the manufacturer’s instructions. Optical density readings were taken at 450 nm on an ELISA reader (THERMO PLATE- TP-READER). The results were obtained after creating standard curves using different concentrations of recombinant cytokines provided by the commercial kits.

### Bone Marrow-derived Macrophages (BMMs)

Two mice from each strain (BALB/c, C57BL/6, TLR-2^-/-^, and TLR-4^-/-^) were used in this experiment. The mice were euthanized by cervical dislocation. The bone marrow cells were washed, processed, and resuspended in cRPMI, adjusted to 10^6^ per mL, and plated in a 24-well plate containing 10 μg/mL recombinant murine granulocyte-macrophage colony stimulating factor (GM-CSF) (eBioscience). After differentiation, the macrophages were resuspended and cultured in a 96-well plate at a concentration of 2 × 10^5^ per well for 24 h before stimulation with the antigens.

### BMM Infection with BCG, rBCG-CMX, and Empty rBCG-pLA71

Bone marrow-derived macrophages (1 × 10^6^ cells/well) were infected with 5x10^6^ CFU of the BCG, rBCG-CMX, or empty rBCG-pLA71 vaccines [MOI 5:1]. Cultures were maintained according to the conditions described above. After 3 h, the supernatants containing non-phagocytosed bacteria were discarded, and the cells were washed twice with PBS at 37°C. After the addition of 500 μL complete RPMI medium without antibiotics, the plate was incubated for 24 h under the same conditions. Subsequently, the culture supernatants were obtained and stored at -20°C for cytokine measurement.

### Immunoblotting

For immunoblotting, macrophages from cultures infected with the BCG, rBCG-CMX, or empty rBCG-pLA71 vaccines were lysed with 50 μL of sterile water after 24 h of culture. A 20-μL aliquot of each lysate was spotted on a nitrocellulose membrane (Trans-Blot-Bio-Rad Laboratories). As a positive control, 20 μL of rCMX (250 μg/mL) was added to the nitrocellulose membrane. The membrane was then blocked with 25 mL of PBS/5% milk. After incubation at 4°C for 18 h with agitation, the membrane was treated with anti-CMX antibody (de Sousa et al., 2012). After 2 h of incubation, the membrane was washed with PBS/0.05% Tween 20 and incubated for 1 h at 37°C with biotinylated anti-IgG1 and anti-IgG2a antibodies (1:15,000-Southern Biotechnology Associates, Inc.). The membrane was then washed again and incubated with Avidin-peroxidase (1:500) in PBS/2% milk for 1 h at room temperature with agitation. After an additional washing step, the membrane was treated with developer buffer containing 0.015% diaminobenzidine (DAB) and 0.03% H_2_O_2_ in PBS, and it was gently shaken and protected from light.

### Indirect Analyses of NF-κB/AP-1 Activity

To evaluate NF-κB/AP-1 activation, RAW-Blue cells (macrophages) that express the SEAP (secreted embryonic alkaline phosphatase) gene under the control of NF-B/AP-1 were used, as described by Zoccal et al. (2014). Activity measurement was performed after 24 h of stimulation with rAg85c and rCMX (2 μg/ml).

### Flow Cytometric Analysis

Macrophages derived from the bone marrow and lung homogenates of BALB/c mice were evaluated using flow cytometry. The lung cells and BMMs were treated with 10% mouse serum for 30 min. After treatment, the cells were washed with 200 μL PBS/azide. After centrifugation, the macrophages were incubated for 30 min with FITC anti-CD206 (Clone MR5D3 – Santa Cruz Biotechnology), anti-CD86 PE (Clone GL1-eBioscience Inc., San Diego, CA, USA), anti-CD11b PerCP (Clone M1/70 – BD Biosciences Pharmingen, San Jose, CA, USA), and anti-F4/80 APC (Clone BM8 - eBioscience, Inc., San Diego, CA, USA) antibodies. Meanwhile, the BMMs were labeled with anti-CD206 FITC (MR5D3 – Santa Cruz Biotechnology), anti-CD86 PE (Clone GL1 – eBioscience Inc., San Diego, CA, USA), anti-MHCII PerCP (Clone M5/114.15.2 – BioLegend), and anti-F4/80 APC (Clone BM8 – eBioscience, Inc. San Diego, CA, USA) antibodies. For intracellular staining, cells were first treated with PermWash (BD PermWash^TM^) and then incubated with anti-TNF-α FITC (Clone MP6-XT22). After the addition of 200 μL PBS/azide and further centrifugation, the cells were treated with PERM FIX (BD Cytofix/Cytoperm^TM^) for 20 min at 2–8°C. They were then washed and resuspended in 200 μL PBS/azide. A total of 50,000 events were acquired using the BD FACS Verse flow cytometer (UFG). Data were analyzed using FlowJo software, version 8.7.

### rBCG-CMX and Empty BCG-pLA71 Immunizations of C57BL/6, TLR-2^-/-^, and TLR-4^-/-^ Mice

Fifteen animals from each mouse lineage were separated into three groups: control, empty BCG-pLA71, and rBCG-CMX (*n* = 5/group). Prior to use, the vaccines were thawed, and the concentrations were adjusted with PBS/0.05% Tween 80 such that each animal received 10^7^ CFU/100 μL by subcutaneous injection in the dorsal region as previously described (da Costa et al., 2014). The vaccine concentrations were confirmed by plating the remaining inoculum on 7H11 agar supplemented with OADC. After immunization, the animals were observed for 3 h to check for signs of apathy or any change in behavior indicating extreme discomfort. If an animal produced signs and symptoms that were incompatible with animal welfare, then a trained veterinarian would humanely euthanize the animal. No animals presented such symptoms during the experiment.

### Splenocyte Preparation

Thirty days after immunization, animals were euthanized by cervical dislocation, and the spleens were collected. Spleens were prepared into single cell suspensions using 70-μm cell strainers (BD Biosciences, Lincoln Park, NJ, USA), and the cells were resuspended in RPMI medium. Erythrocytes were lysed with lysis solution (0.15 M NH_4_Cl, 10 mM KHCO_3_), and the cells were washed and resuspended in RPMI supplemented with 20% fetal calf serum, 0.15% sodium bicarbonate, 1% L-glutamine (200 mM; Sigma–Aldrich-Brazil, São Paulo), 1% non-essential amino acids (Sigma–Aldrich), and 1% penicillin/streptomycin (1,000 U/mL GIBCO). Cells were counted in a Neubauer chamber, and the concentration was adjusted to 1 × 10^6^ cells/mL.

### Th1 and Th17 CMX-specific Response of Splenocytes from C57BL/6, TLR-2^-/-^, and TLR-4^-/-^ Mice

In a 96-well culture plate (Cell/Wells^TM^), 2 × 10^5^ splenocytes (200 μL) were cultivated with recombinant CMX (10 mg/mL), ConA (positive control), or with media alone (no stimulus) in a 5% CO_2_ incubator at 37°C for 4 h. Monensin (3 mM; eBioscience) was then added to the wells, and the cultures were incubated another 4 h. Cells were treated with PBS containing 0.1% sodium azide for 30 min at room temperature. After centrifugation, the cells were stained with anti-CD4 FITC (BD PharMingen, clone RM4-5) for 30 min. Cells were then permeabilized with Perm Fix/Perm Wash (BD PharMingen), washed with PBS containing 0.1% sodium azide, and stained with anti-IFN-γ APC (eBioscience; clone: XMG1.2) to assess the Th1 cell profile. For Th17 analysis, cells were stained with anti-IL-17A PerCP (eBioscience, clone: eBio17B7) for 30 min. The cell suspensions were stained using 25 μL of a monoclonal antibody mixture containing both anti-IFN-γ and anti-IL-17 antibodies. A total of 50,000 events were acquired using a BD FACS Verse (Universidade Federal de Goiás- UFG) flow cytometer. The acquired data were analyzed using FlowJo 8.7 software. Lymphocytes were selected based on their size (forward scatter, FSC) and granularity (side scatter, SSC). CD4^+^ T cells were gated and evaluated in a dot plot using the fluorescence for CD4 (FITC) and the cytokine fluorescence [IFN-γ (APC) or IL-17 (PerCP)]. To define the negative population, an isotype Rat IgG2a antibody was used (APC or PerCP). The specific immune responses were determined by subtracting the result of the splenocytes cultured without stimulation from the responses with CMX recombinant antigen.

### Statistical Analysis

Data were tabulated and analyzed using Microsoft Office Excel 2011 and Prism software (version 5.0c, GraphPad). The results are presented as the means and standard deviations for each experimental group. The results using recombinant proteins as stimuli were evaluated by multimetric tests using a one-way ANOVA followed by comparison of each experimental group with the negative control group (medium) using Dunn’s test. *P* < 0.05 was considered statistically significant. All experiments were repeated three times.

## Results

### rBCG-CMX Vaccine Promotes Increased Pulmonary Macrophage Population

**FIGURE 1:**
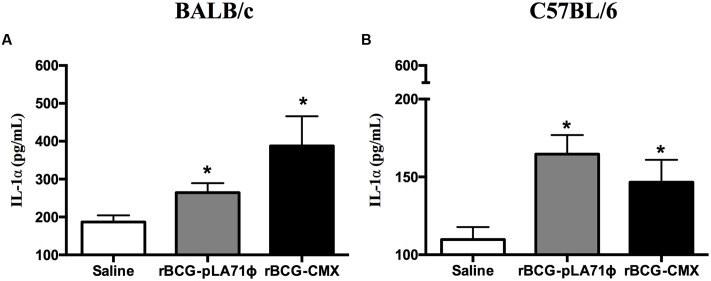
**IL-1α production by lung homogenates from Bacillus Calmette–Guérin (BCG) vaccinated mice.** Mice were intranasally vaccinated with BCG-pLA71 or rBCG-CMX. The animals were euthanized 4 days after immunization, and the production of IL-1α was evaluated in lung homogenates. BALB/c mice **(A)**; C57BL/6 mice **(B)**. ^∗^*p* < 0.05 difference between the rBCG-CMX or BCG-pLA71 group and the saline group. No difference was observed between the BCG-pLA71 and rBCG-CMX groups. A total of five mice were used per group. The experiments were repeated three times.

**FIGURE 2:**
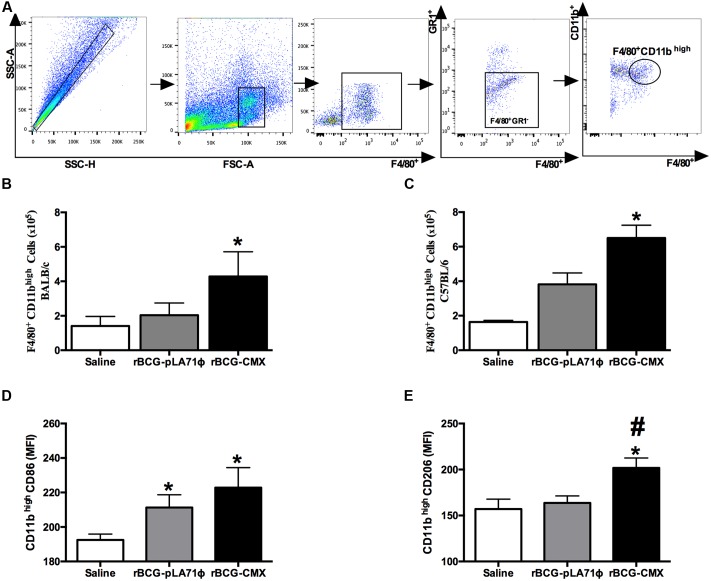
***In vivo* induction of macrophage profile.** Mice were intranasally vaccinated with BCG-pLA71 or rBCG-CMX. Immunization was performed using 10^7^ CFU of vaccine per mouse. The animals were euthanized 4 days after immunization, and flow cytometry was performed with lung homogenates to observe macrophage activity. **(A)** Dot plot of cytometry for F4/80^+^CD11b^high^. **(B)** Numbers of F4/80^+^CD11b^high^ macrophages in BALB/c mice. **(C)** Numbers of F4/80^+^CD11b^high^ macrophages in C57BL/6 mice. **(D)** Median intensity of fluorescence (MFI) of F4/80^+^CD11b^high^ macrophages expressing CD86. **(E)** Median intensity of fluorescence (MFI) of F4/80^+^CD11b^high^ macrophages expressing CD206. ^∗,#^*p* < 0.05. ^∗^Difference between the rBCG-CMX group and the saline group. ^#^Difference between the rBCG-CMX group and the BCG-pLA71 group. A total of five mice were used per group. The experiments were repeated three times.

IL-1α is responsible for the proliferation of alveolar macrophages and the induction of CD11b expression (Gonzales-Juarrero et al., 2003; Huaux et al., 2015). BALB/c and C57BL/6 mice were intranasally infected/vaccinated (Lyadova et al., 2001), and after 4 days, the macrophages and lung homogenates were evaluated. Lung homogenates from rBCG-CMX-vaccinated BALB/c and C57BL/6 mice presented higher levels of IL-1α (**Figure 1**, *p* < 0.05). Because IL-1α induction was achieved by intranasal inoculation with the vaccines, we asked whether the vaccines participated in increasing the levels of the co-stimulatory molecules CD86 and CD206 in the macrophages of vaccinated mice. The lungs of mice vaccinated with rBCG-CMX had a higher number of macrophages (F4/80^+^CD11b^high^) compared to mice vaccinated with empty BCG-pLA71 (**Figures 2A–C**; *p* < 0.05). The F4/80^+^CD11b^high^ macrophages induced by rBCG-CMX or empty BCG-pLA71 expressed similar levels of CD86 (**Figure 2D**). However, F4/80^+^CD11b^high^ macrophages presented higher expression of CD206 compared with either the empty BCG-pLA71 vaccine or saline alone (**Figure 2E**; *p* < 0.05). The rBCG-CMX vaccine thus promoted an increase in macrophages with high CD86 and CD206 expression in the lungs of infected animals, indicating a difference in activation from the immune response induced by empty BCG-pLA71.

**FIGURE 3:**
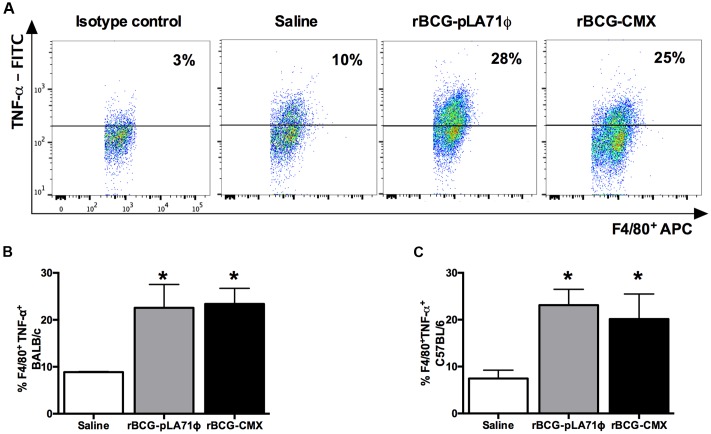
**Macrophages in the lung expressing TNF-α upon BCG-CMX and BCG-pLA71 vaccination.** Mice were intranasally vaccinated with BCG-pLA71 or rBCG-CMX. Immunization was performed using 10^7^ CFU of vaccine per mouse. The animals were euthanized 4 days after immunization, and flow cytometry of lung homogenates was performed to observe macrophages expressing TNF-α. **(A)** Gate strategy of C57BL/6 mice; **(B)** BALB/c mice; **(C)** C57BL/6 mice. A total of five mice were used per group. The experiments were repeated three times. ^∗^Difference between rBCG-pLA71/rBCG-CMX group and saline group.

Thus, we decided to determine whether BCG-CMX also differentially induced TNF-α. The numbers of macrophages (F4/80+) expressing TNF-α were similar in the lung homogenates of BCG-pLA71 and BCG-CMX-vaccinated animals (**Figures 3A–C**).

### The rCMX Protein Induces the Production of Cytokines in BMMs

**FIGURE 4:**
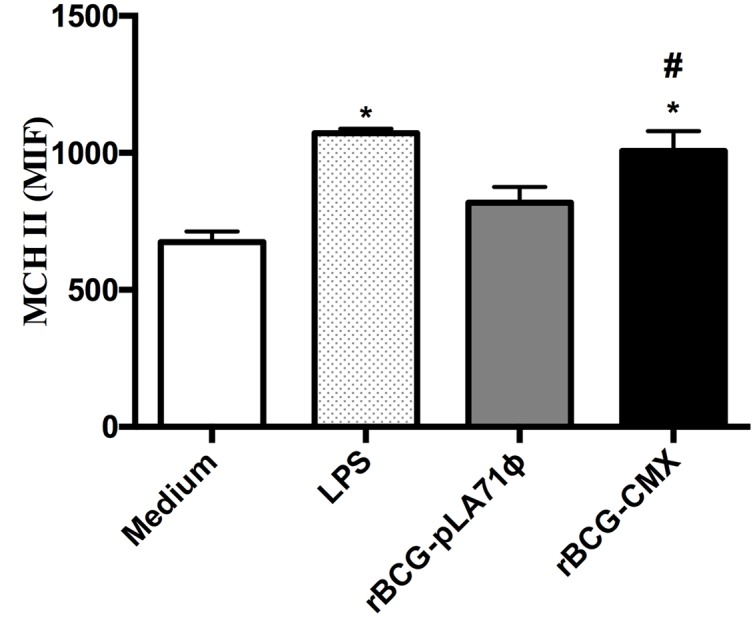
**Macrophage infected by rBCG-CMX vaccine increases MHC-II.** Bone marrow-derived macrophages were infected with BCG or rBCG-CMX (MOI 5) for 3 h. After this time, excess non-phagocytosed bacteria were removed. After 24 h of culture, the macrophages were analyzed by flow cytometry. F4/80 positive cells were evaluated for the mean intensity of fluorescence (MFI) of MHC-II expression. ^∗^Difference between the rBCG-CMX group and the saline group. ^#^Difference between the rBCG-CMX group and the BCG group, *p* < 0.05. The experiments were repeated three times in quadruplicate.

**FIGURE 5:**
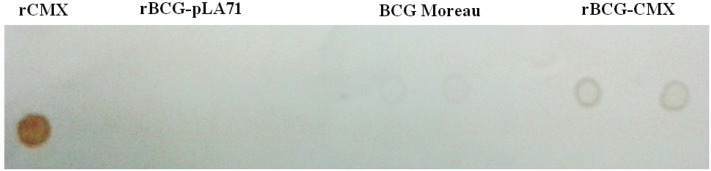
**Recombinant CMX protein is expressed in macrophages infected with rBCG-CMX vaccine.** Bone marrow-derived macrophages were infected with BCG or rBCG-CMX (MOI 5) for 3 h. After this period, excess non-phagocytosed bacteria were removed. After 24 h of culture, the macrophages were lysed, and dot blots were performed to detect rCMX protein expression. As a positive control, purified rCMX was added. As negative controls, BCG containing empty vector (rBCG-pLA71) and BCG-Moreau vaccines were added. The experiments were repeated three times in quadruplicate.

Some Mtb proteins are individually able to activate the immune response in macrophage models, inducing either a pro- or an anti-inflammatory response (Kim et al., 2012; Kumar et al., 2013; Tiwari et al., 2014). In the present study, the rBCG-CMX vaccine promoted greater expression of MHC-II in infected macrophages compared with empty BCG-pLA71 (**Figure 4**; ^∗^*p* < 0.05). Therefore we proposed that the rCMX protein could activate inflammation, thereby promoting the induction of better vaccine protection. We first studied the expression of the rCMX protein within BMMs, and expression was confirmed by dot blots of infected macrophages (**Figure 5**).

**FIGURE 6:**
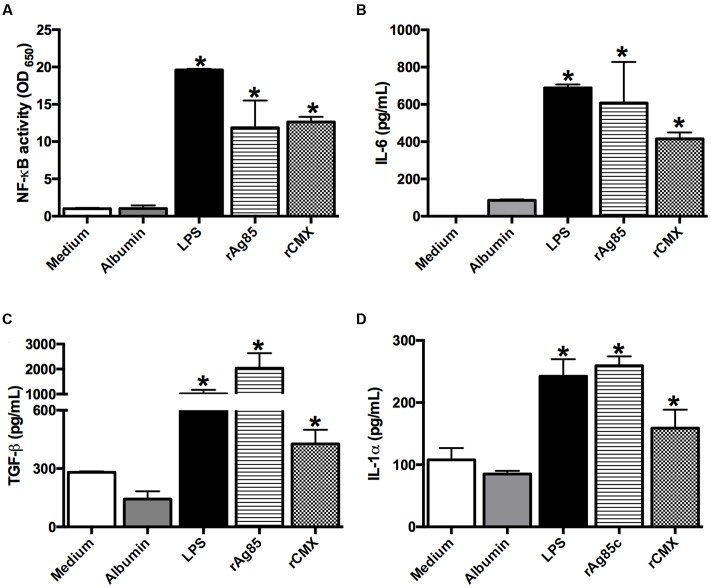
**Production of cytokines by macrophages stimulated with the rAg85c and rCMX proteins.** RAW-Blue cells (RAW 264.7) were stimulated with 20 μg/mL of recombinant proteins for 24 h. Supernatants were obtained for the indirect measurement of NF-κB activity **(A)**. Macrophages derived from the bone marrow of BALB/c mice were cultured with 20 μg/mL recombinant proteins for 24 h. After this period, the collected supernatant was used to measure the levels of IL-6 **(B)**, TGF-β **(C)**, and IL-1α **(D)** cytokines. ^∗^*p* < 0.05 difference between stimuli and the medium. The experiments were repeated three times in quadruplicate.

Additionally, we observed that rCMX was able to induce the activation of NF-κB (**Figure 6A**); consequently, the production of IL-6 was evaluated in macrophages. BMMs from BALB/c mice were stimulated with rCMX or Ag85c for 24 h (**Figure 6B**; *p* < 0.05), and both proteins induced IL-6 production. As stated previously, the rBCG-CMX vaccine induces increased TGF-β and IL-1α production in the lungs of BALB/c mice. We therefore asked whether rCMX was capable of inducing macrophages to produce these cytokines. The results demonstrate that both rAg85c and rCMX induced TGF-β and IL-1α in the BMMs from BALB/c mice (**Figures 6C,D**; *p* < 0.05).

A vaccine should be able to induce similar responses across a varied genetic background (Garcia-Pelayo et al., 2015). The mouse strains C57BL/6 and BALB/c are known to have genetic differences that could affect the induction of Mtb responses (Paula et al., 2011). Furthermore, after vaccination with BCG, protective immune response induction has been shown to differ between these two models (Paula et al., 2011; Sérgio et al., 2015). Thus, we asked whether the rCMX protein could activate macrophages from C57BL/6 mice.

**FIGURE 7:**
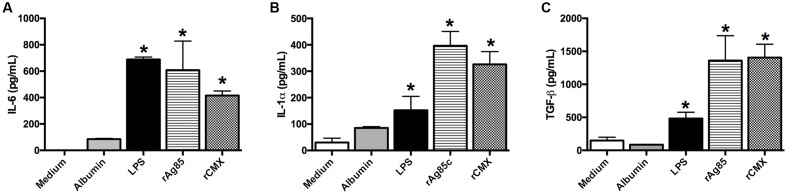
**Production of cytokines by BMMs from C57BL/6 mice stimulated with rAg85c and rCMX proteins.** Macrophages derived from the bone marrow of C57BL/6 mice were cultured with 20 μg/mL recombinant proteins for 24 h. After this period, the collected supernatant was subjected to analyses of IL-6 **(A)**, IL-1α **(B)**, and TGF-β **(C)** cytokines. ^∗^*p* < 0.05 difference between stimuli and the medium. The experiments were repeated three times in quadruplicate.

For this purpose, BMMs from C57BL/6 mice were stimulated with the rAg85c and rCMX proteins for 24 h. IL-6 induction was observed for both proteins (**Figure 7A**; *p* < 0.05). rAg85c and rCMX also induced the production of IL-1α (**Figure 7B**; *p* < 0.05) and TGF-β (**Figure 7C**; *p* < 0.05).

**FIGURE 8:**
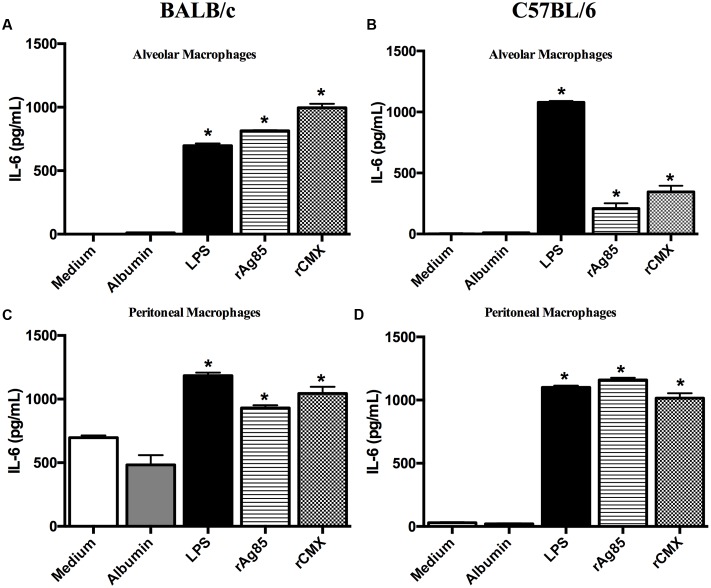
**Production of IL-6 by alveolar and peritoneal macrophages from BALB/c and C57BL/6 mice after stimulation with rAg85c and rCMX proteins.** Alveolar and peritoneal macrophages from BALB/c and C57BL/6 mice were obtained and cultured with 20 μg/mL recombinant proteins for 24 h. After this period, the collected supernatant was used to measure the level of IL-6. **(A)** Alveolar macrophages from BALB/c; **(B)** alveolar macrophages from C57BL/6 mice; **(C)** peritoneal macrophages from BALB/c mice; and **(D)** peritoneal macrophages from C57BL/6 mice. The experiments were repeated three times in quadruplicate. ^∗^*p* < 0.05 difference between stimuli and the medium.

Bone marrow-derived macrophages are a good model for eliminating natural activation bias, but they do not reliably reflect infection or immunization. To better simulate the mucosal environment, we used alveolar and non-stimulated peritoneal macrophages from both mouse strains. These macrophages could allow us to evaluate the behavior of the cells of the primary infection site and the peripheral response. The results show that the Mtb recombinant proteins induced IL-6 production in the alveolar and peritoneal macrophages of BALB/c and C57BL/6 mice (**Figure 8**; *p* < 0.05).

### TLR-4 Appears to Participate in the Induction of IL-6 by rAg85c and rCMX

Given the context in which these proteins stimulate the immune response, we asked whether they were recognized by innate immune response receptors. In the context of TB, TLR-2 and TLR-4 are implicated in host cell interactions with Mtb (Kim et al., 2012; Kumar et al., 2013). Due to the increased production of IL-6 inflammatory mediators of the NF-κB activity, we used BMMs from TLR-2^-/-^ and TLR-4^-/-^ mice to explore whether TLR-2 and TLR-4 are involved in recognizing the rAg85c and rCMX proteins.

**FIGURE 9:**
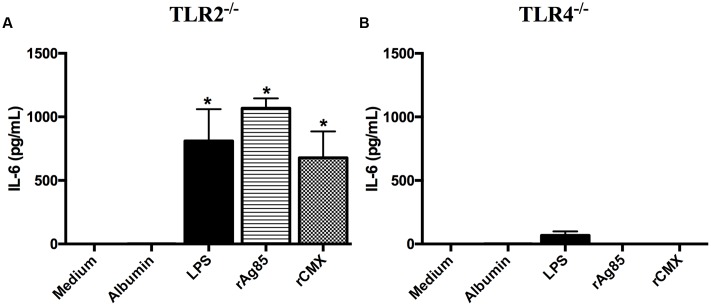
**TLR-4 is involved in the recognition of rAg85c and rCMX by BMMs.** Macrophages derived from the bone marrow of TLR-2^-/-^ and TLR-4^-/-^ mice were cultured with 20 μg/mL recombinant proteins for 24 h. After this period, IL-6 production was evaluated from TLR-2^-/-^ macrophages **(A)** or from TLR-4^-/-^ macrophages **(B)**. The experiments were repeated three times in quadruplicate. ^∗^*p* < 0.05 difference between the stimuli and the medium.

Twenty-four hours after stimulating BMMs from TLR-2^-/-^ mice with rAg85c and rCMX proteins, IL-6 production occurred at a similar level as in control BMMs (**Figure 9A**; *p* < 0.05). However, IL-6 production was reduced in the BMMs of TLR-4^-/-^ mice (**Figure 9B**). These results demonstrate that the interaction of rAg85c and rCMX proteins with TLR-4 may be necessary for IL-6 production.

### Lack of CMX-Specific Th1 and Th17 Induction after TLR-2^-/-^ and TLR-4^-/-^ Mouse Vaccination with rBCG-CMX

Because the recombinant vaccine induced different responses and because we have shown that rCMX stimulates NF-κB and appears to involve TLR signaling, we asked whether TLR-2 or TLR-4 receptors were important for the development of the specific Th1 and Th17 responses required for rBCG-CMX protection (da Costa et al., 2014).

**FIGURE 10:**
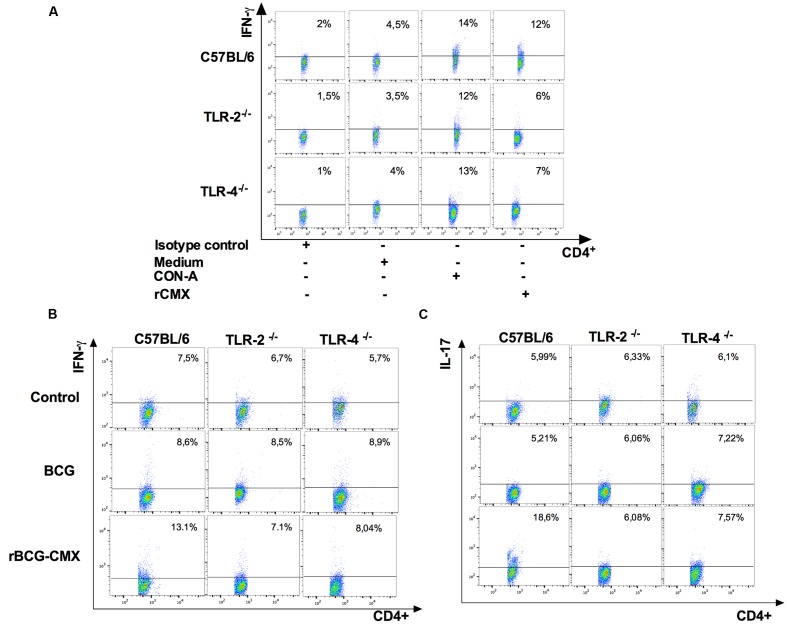
**Gate strategy for levels of CD4^+^IFN-γ^+^ and CD4^+^IL-17^+^ T cells induced by *ex vivo* stimulation with recombinant CMX.** **(A)** Lymphocytes were selected based on their size and granularity. CD4^+^IgG^+^ (Isotype control) and CD4^+^IFN-γ^+^ from C57BL/6, TLR-2^-/-^, and TLR-4^-/-^ immunized mice were cultured with medium, CON-A or rCMX. **(B,C)** Lymphocytes were selected based on their size and granularity, the antigen-specific CD4^+^IFN-γ^+^ **(B)** and CD4^+^IL-17^+^ T cells **(C)** were analyzed based on their fluorescence.

**FIGURE 11:**
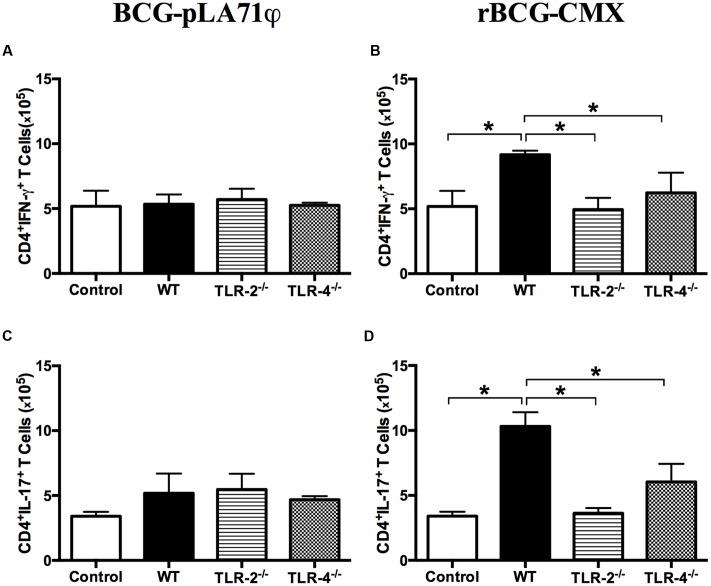
**Levels of CD4^+^IFN-γ^+^ and CD4^+^IL-17^+^ T cells induced by *ex vivo* stimulation with recombinant CMX.** Thirty days after vaccination, splenocytes from C57BL/6, TLR-2^-/-^, and TLR-4^-/-^ non-immunized (control) or mice immunized with BCG or with rBCG-CMX were stimulated with recombinant CMX. CD4^+^IFN-γ^+^ T cells from mice vaccinated with BCG-pLA71 **(A)** or rBCG-CMX **(B)**. CD4^+^IL-17^+^ T cells from mice vaccinated with BCG-pLA71 **(C)** or rBCG-CMX **(D)**. ^∗^*p* < 0.05 statistical difference between groups. This is a representative experiment using three mice per group. The experiments were repeated three times.

C57BL/6, TLR2^-^/^-^, and TLR4^-^/^-^ mice were vaccinated with rBCG-CMX or empty BCG-pLA71. After 30 days, the specific responses to CMX were evaluated in CD4^+^ T splenocytes. C57BL/6 mice vaccinated with rBCG-CMX doubled the number of Th1 subtype (CD4^+^ IFN-γ^+^) T cells, but this was not observed in the splenocytes from TLR2^-^/^-^ and TLR4^-^/^-^ rBCG-CMX-vaccinated mice (**Figures 10**, **11B**). Similarly, C57BL/6 mice presented significantly more CMX-specific Th17 cells, whereas rBCG-CMX vaccination of TLR2^-^/^-^ and TLR4^-^/^-^ mice did not induce a specific CD4^+^IL-17^+^ T cell increase in the spleen (**Figures 10**, **11D**; *p* < 0.05). The Th1 and Th17 CMX-specific responses were not observed in the splenocytes from animals vaccinated with empty BCG-pLA71 (**Figures 11A,C**).

## Discussion

Previous studies have shown that the rBCG-CMX vaccine induces both Th1 and Th17 responses, and both responses are important in controlling Mtb infection. In the present study, we propose that the expression of the rCMX protein by the rBCG vaccine (rBCG-CMX) activates the innate immune response and modulates the response of this vaccine, promoting the induction of the Th1 and Th17 responses. These results demonstrate that macrophages activated by the rBCG-CMX vaccine are more numerous and have greater CD86 and CD206 expression accompanied by the production of IL-1α. The rCMX protein was able to induce IL-1α, IL-6, and TGF-β production. The absence of TLR-4 or TLR-2 abrogates the induction of the Th1 and Th17 CMX-specific responses by rBCG-CMX vaccination.

The mucosa contains important cells, such as macrophages, which are essential in the production of innate immune memory after vaccination (Yoshida et al., 2015). In Mtb infection, the first induced response begins primarily on the surface of the respiratory mucosa; therefore, the first line of defense should be produced at the pathogen infection site to promote a better protective response (Giri and Khuller, 2008). An alternative method of evaluating the ability of the rBCG-CMX vaccine to activate the innate immune response is therefore to evaluate pulmonary macrophages after infection with the vaccine.

After 4 days of infection, the rBCG-CMX and BCG vaccines induced greater IL-1α production than the saline group (**Figure 1B**). Huaux et al. (2015) observed that IL-1α is responsible for the proliferation of CD11b^low^ alveolar macrophages and the activation of CD11b^high^ macrophages (Huaux et al., 2015). This observation suggests that the rBCG-CMX and BCG vaccines may induce greater proliferation of the macrophages after infection. These macrophages migrate to the lung tissue and are active in granuloma formation (Huaux et al., 2015). However, after infection, only the rBCG-CMX vaccine was shown to induce a greater number of F4/80^+^CD11b^high^ macrophages in the lung, indicating that this vaccine has a good potential to promote macrophage recruitment to the infection site. In our results using the rBCG-CMX vaccine, F4/80^+^CD11b^high^ macrophages had similar CD86 expression and greater CD206 expression compared to the BCG-Moreau vaccine. The expression of CD206 also characterizes the M2 macrophages, which are known as anti-inflammatory macrophages (Kambara et al., 2015). Our results corroborate the study by Mirza and Koh (2011), who showed that TGF-β is produced concomitantly with the induction of CD206 expression (Mirza and Koh, 2011). These cells are important during the activation of the adaptive immune response because the co-stimulatory molecules CD86 and CD206 and phagocytosis receptors are strongly associated with the presentation of antigens to T lymphocytes (Edwards et al., 2006; Azad et al., 2014).

These findings led us to hypothesize that the rCMX protein could modulate the response induced by the BCG vaccine, modifying and improving its ability to activate the immune response. The rCMX protein consists of the immunodominant epitopes of rAg85c and rMPT51 and full-length rHspX protein (de Sousa et al., 2012). When expressed by the rBCG vaccine (rBCG-CMX), the rCMX protein modifies the immune response induced by empty BCG-pLA71 (BCG with the empty plasmid). Therefore, we suggest that the rCMX protein modulates the response of the BCG vaccine and the macrophage response. Several studies have shown that some Mtb proteins are able to activate the innate immune response in macrophages and dendritic cells to interact with TLRs (Kim et al., 2012; Xu et al., 2015). We therefore verified the pro-inflammatory capacity of Ag85c and rCMX, which allowed us to infer the immunomodulatory effects of these proteins. We observed that the rAg85c and rCMX proteins activated NF-κB, highlighting the ability of these proteins to induce a pro-inflammatory response (Pagliari et al., 2000). It is important to note that the other proteins that compose rCMX (HspX and MPT51) were tested, as was a non-related recombinant protein, and no macrophage stimulation was observed. This hypothesis is supported by the fact that rAg85c has been shown to exhibit powerful pro-inflammatory activity.

The C57BL/6 and BALB/c strains are known to differ in their adaptive immune response induction after BCG vaccination or Mtb infection. However, this difference does not affect the control of infection by the two models (Paula et al., 2011; Sérgio et al., 2015). Regardless of the model utilized, rAg85c and rCMX induced macrophage activation, suggesting that these proteins are able to activate the immune response in BMMs from both models.

Because the observed activation of inflammation may be related to the BMM response profile, we evaluated this response in alveolar and peritoneal macrophages, which best represent a mucosal microenvironment. Although the induction of IL-6 production by rCMX varied between the two models, IL-6 cytokine production was maintained between the different macrophage profiles. The innate immune response produced by the rCMX protein, including the induction of pro- and anti-inflammatory cytokines such as IL-6, IL-1α, and TGF-β, may have promoted the induction of Th17 responses *in vivo* and may have contributed to the greater protection induced by BCG when rCMX was used as a booster (da Costa et al., 2014).

Xu et al. (2015) have shown that PPE57 from Mtb is capable of regulating the immune response of the host by interacting with TLR-2. In the present study, we demonstrated that rAg85c and rCMX may have interacted with TLR-4 to promote IL-6 induction *in vitro*. A similar response was observed with the Rv0652 protein of Mtb, which induced a TLR-4-dependent pro-inflammatory immune response by stimulating BMMs and RAW 264.7 macrophages (Kim et al., 2012). Furthermore, our results demonstrate that the induction of the Th1 and Th17 adaptive immune response by rBCG-CMX was somehow dependent on TLR-2 and TLR-4 receptors in vaccinated mice (**Figure 11**).

Among the difficulties and limitations of the present work, we should mention the potential contamination of the proteins with LPS, and to solve this problem, LPS was removed from the recombinant proteins using LPS extraction kits (Toxin Removal Kit), which are currently being used in Mtb protein testing (Li et al., 2014). We used TLR-4 KO and TLR-2 KO mice, and it might be thought that these mice have increased susceptibility to mycobacterial infection. However, it has been shown previously that double TLR-2/TLR-4 KO mice are not more susceptible to BCG infection; they are able to produce a normal and specific T helper cell response and control BCG infection (Nicolle et al., 2004).

These results allow us to conclude that the rCMX protein modulates the innate immune response by activating macrophages and inducing IL-6, IL-1α, and TGF-β. Further studies should be performed to confirm the interaction between rCMX and TLR-4 and to study the importance of this interaction in inducing CMX-specific Th1 or Th17 cells.

## Author Contributions

Experimental design and set up: AJ-K and AK. Experimental development and data analyses: AJ-K, AK, AdC, DR, BS, and KZ. Grant PIs that secured funding for reagents, materials, and analysis tools for all experiments: AJ-K, AK, and LF. Critical discussion and writing of the manuscript: AJ-K, AK, AdC, DR, BS, KZ, and LF. Cytometry experiments and analysis: AdC.

## Conflict of Interest Statement

The authors declare that the research was conducted in the absence of any commercial or financial relationships that could be construed as a potential conflict of interest.
